# Associations between immune cell phenotypes and lung cancer subtypes: insights from mendelian randomization analysis

**DOI:** 10.1186/s12890-024-03059-w

**Published:** 2024-05-16

**Authors:** Jin-Min Zheng, Chen-Xi Lou, Yu-Liang Huang, Wen-Tao Song, Yi-Chen Luo, Guan-Yong Mo, Lin-Yuan Tan, Shang-Wei Chen, Bai-Jun Li

**Affiliations:** 1 Department of Surgery, Guangxi Medical University, Nanning, Guangxi China; 2 Department of Surgery, Guangxi University of Chinese Medicine, Nanning, Guangxi China; 3 Department of Surgery, Youjiang Medical University For Nationalities, Baise, Guangxi China; 4 Department of thoracic surgery, Guangxi Academy of Medical Sciences and the People’s Hospital of Guangxi Zhuang Autonomous Region, Nanning, Guangxi China; 5 Department of thoracic surgery, Guilin Medical University, Guilin, Guangxi China; 6 Department of thoracic surgery, Tumor Hospital of Guangxi Medical University, Nanning, Guangxi China

**Keywords:** Mendelian randomization analysis, Lung squamous cell carcinoma, Lung adenocarcinoma, Immune cell

## Abstract

**Introduction:**

Lung cancer is a common malignant tumor, and different types of immune cells may have different effects on the occurrence and development of lung cancer subtypes, including lung squamous cell carcinoma (LUSC) and lung adenocarcinoma (LUAD). However, the causal relationship between immune phenotype and lung cancer is still unclear.

**Methods:**

This study utilized a comprehensive dataset containing 731 immune phenotypes from the European Bioinformatics Institute (EBI) to evaluate the potential causal relationship between immune phenotypes and LUSC and LUAD using the inverse variance weighted (IVW) method in Mendelian randomization (MR). Sensitivity analyses, including MR-Egger intercept, Cochran Q test, and others, were conducted for the robustness of the results. The study results were further validated through meta-analysis using data from the Transdisciplinary Research Into Cancer of the Lung (TRICL) data. Additionally, confounding factors were excluded to ensure the robustness of the findings.

**Results:**

Among the final selection of 729 immune cell phenotypes, three immune phenotypes exhibited statistically significant effects with LUSC. CD28 expression on resting CD4 regulatory T cells (OR 1.0980, 95% CI: 1.0627–1.1344, *p* < 0.0001) and CD45RA + CD28- CD8 + T cell %T cell (OR 1.0011, 95% CI: 1.0007; 1.0015, *p* < 0.0001) were associated with increased susceptibility to LUSC. Conversely, CCR2 expression on monocytes (OR 0.9399, 95% CI: 0.9177–0.9625, *p* < 0.0001) was correlated with a decreased risk of LUSC. However, no significant causal relationships were established between any immune cell phenotypes and LUAD.

**Conclusion:**

This study demonstrates that specific immune cell types are associated with the risk of LUSC but not with LUAD. While these findings are derived solely from European populations, they still provide clues for a deeper understanding of the immunological mechanisms underlying lung cancer and may offer new directions for future therapeutic strategies and preventive measures.

**Supplementary Information:**

The online version contains supplementary material available at 10.1186/s12890-024-03059-w.

## Introduction

Lung cancer is the second most prevalent cancer worldwide, where non-small cell lung cancer (NSCLC) is the principal pathological phenotype [1]. NSCLC encompass two major subtypes, lung squamous cell carcinoma (LUSC) and lung adenocarcinoma (LUAD), based on their cell origin, morphology, and biological characteristics [2]. Research recognizes a number of risk factors that contribute to lung cancer, such as smoking, past pulmonary diseases, air pollutants, and occupational carcinogens [3, 4]. The identification and screening of potentially alterable risk factors are essential to lower the incidence of lung cancer, thereby aiding in its early diagnosis and treatment.

Research has reported that the inflammatory immune mechanism is an important feature of the tumor immune microenvironment (TIME) and is associated with poor prognosis in cancer [5, 6]. The location, type, density, and functional status of immune cells constitute the immune structure of the TIME, which varies among patients with NSCLC [7]. Immune cells may exhibit dual roles in both anti-tumor and pro-tumor effects. For example, CD8^+^ T cells and natural killer (NK) cells mediate anti-tumor responses, showing better overall survival, disease-free survival, and progression-free survival [8, 9]. Conversely, regulatory T cells (Tregs) can secrete inhibitory cytokines such as transforming growth factor-beta (TGF-β) and interleukin-10 (IL-10). By inhibiting anti-tumor responses of helper T cells (Th1) and attracting activated Th2 cells, Tregs promote the progression of lung cancer through angiogenesis and immune suppression [10–12]. Observational research revealed that increased proportions of regulatory T cells and M2 macrophage indicated poor survival in advanced NSCLC patients [13]. Gaudreau et al. found that neoadjuvant chemotherapy is associated with increased infiltration of cytotoxic CD8^+^ T cells and CD20^+^ B cells, promoting anti-tumor immunity through changes in the phenotype of cytotoxic and memory CD8^+^ and CD4^+^ T cells [14]. McGrail et al. suggested that the count of CD8^+^ T cells in lung cancer is positively correlated with neoantigen load, with a significantly higher objective response rate in tumors with high tumor mutation burden (TMB) compared to those with low TMB [15]. Devi-Marulkar et al. observed high expression of TIGIT and CTLA-4 in TIL-Tregs within tertiary lymphoid structures (TLS) and non-TLS areas, indicating their involvement in the immune inhibitory mechanisms of lung tumors [16]. Moreover, chronic inflammation is also closely associated with the development of lung cancer, manifested by the infiltration and accumulation of inflammatory cells and the buildup of pro-inflammatory factors [17]. Despite extensive research on the genomic landscape of lung cancer, new factors that contribute to lung cancer are still being discovered [18, 19].

Mendelian randomization (MR) is an analytical method used for epidemiological causal inference, based on Mendel’s law of independent assortment [20]. A recent MR analysis demonstrated that C-C motif chemokine 27 (CCL27) levels in circulation are positively associated with the risk of lung cancer, whereas interleukin-18 levels are inversely associated with such risk [21].

While many pieces of evidence suggest that immune cells play a role in lung cancer risk and outcome, the systemic analysis of immune cells in this context remains undefined, making further comprehensive investigations necessary. In this study, we aimed to comprehensively explore the causal effects of 731 immune cells on lung cancer through a genome-wide association study (GWAS) pooled data using two-sample Mendelian randomization approach. To enhance persuasion, we also performed replication and meta-analysis on another lung cancer cohort. This is the most comprehensive study of the causal link of immune cells with lung cancer, and we hope that our results will provide a more informed assessment of lung cancer risk regarding immune cells.

## Materials and methods

### Study design

We utilized MR methodology to investigate the potential causal relationship between 731 immune cells and LUSC and LUAD. In our research, MR uses SNPs as “instrumental variables”(IV). For MR effect estimation to be robust, and for SNPs to serve as IVs. These assumptions must be as follows: (1) Assumption of Association: Genetic variants are strongly correlated with the exposure factor. (2) Assumption of Independence: Genetic variants are independent of confounding factors. (3) Assumption of Exclusivity: Genetic variants can only affect the outcome through the exposure factor [22]. Based on this, we make three assumptions:

#### Assumption 1

Gene SNPs are closely associated with immune cells.

#### Assumption 2

Gene SNPs are not associated with the outcome variable lung cancer or other confounding factors.

#### Assumption 3

**Fig. 1:**
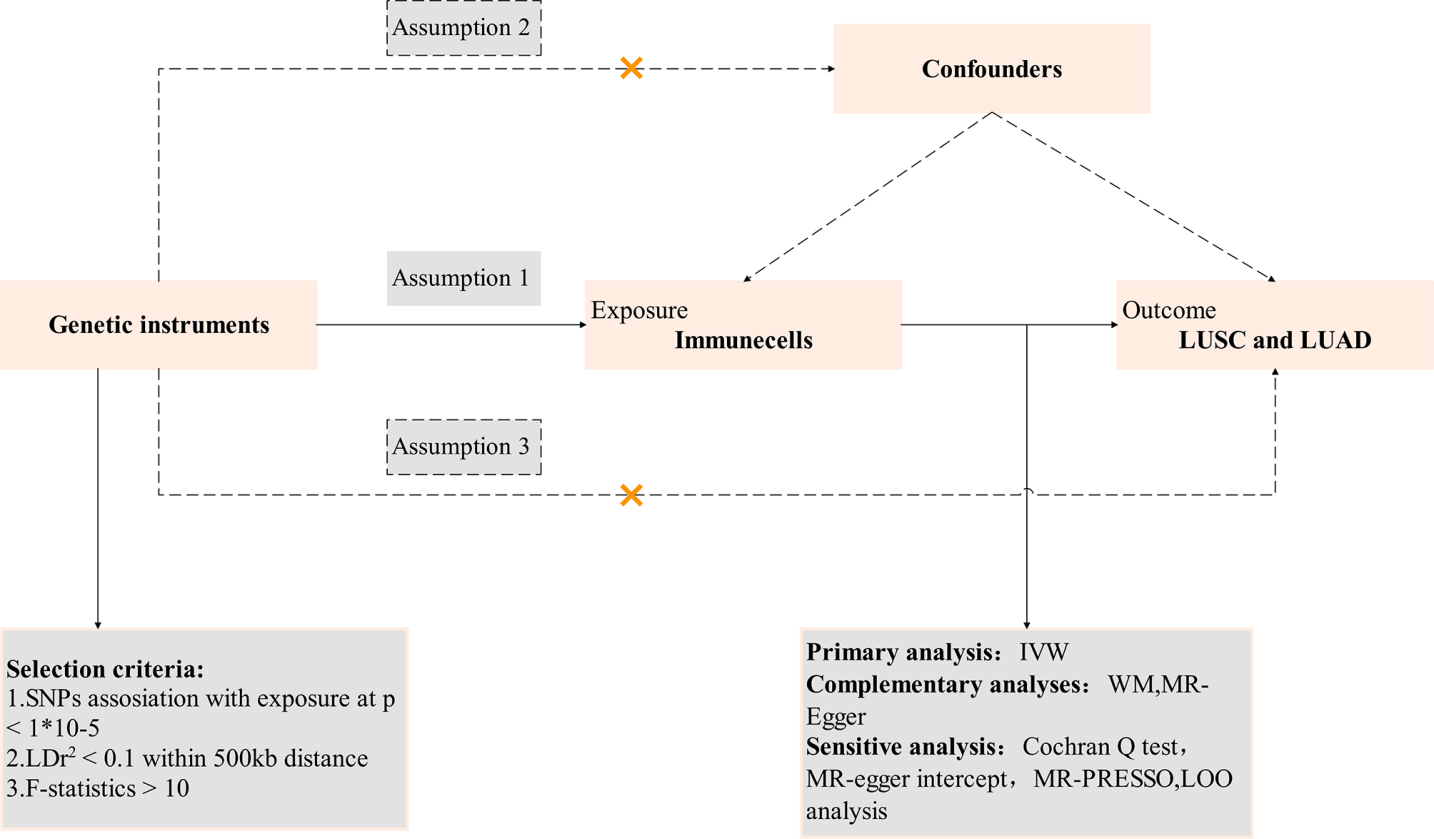
Overview of this Mendelian randomization (MR) analysis. Assumption 1, genetic instruments are strongly associated with the exposures of interest; Assumption 2, genetic instruments are independent of confounding factors; Assumption 3, genetic instruments are not associated with outcome and affect outcome only via exposures. *LUSC* lung squamous cell carcinoma; *LUAD* lung adenocarcinoma; *IVW* inverse variance weighted; *LD* linkage disequilibrium; *LOO analysis* leave-one-out analysis; *MR-PRESSO* MR‐Pleiotropy RESidual sum and outlier; *SNPs* single nucleotide polymorphisms; *WM* weighted median

Gene SNPs only affect lung cancer through their influence on immune cells and cannot affect lung cancer through other pathways (Fig. 1).

### STROBE-MR (strengthening the reporting of observational studies in epidemiology using mendelian randomisation) checklist

This study was guided by the STROBE-MR guidelines. This article adheres to the STROBE-MR checklist for reporting. (Supplementary STROBE-MR-checklist)

### Genome-wide association study (GWAS) data sources for LUSC and LUAD

GWAS summary data for lung cancer (including LUSC and LUAD) were download from GWAS Catalog (https://www.ebi.ac.uk/gwas/) and the GWAS Catalog accession number is GCST004750 (LUSC) and GCST004744 (LUAD). The study performed a GWAS on 129,809 European individuals (*N*_case_=18,699, *N*_control_=111,110), with approximately 156,688,129 variants analyzed after quality control and imputation. Genetic information for lung cancer subtypes (LUSC: *N*_case_=7,426 and *N*_control_=55,627; LUAD: *N*_case_=11,273 and *N*_control_=55,483) were utilized for subgroup analysis. More detailed information about the GWAS data can be obtained from the study of James D McKay et al. [23]. The GWAS data for LUSC and LUAD mentioned above were used for preliminary analysis.

### Immunity-wide GWAS data sources

We collected statistical data for each immune feature from the GWAS catalog, referencing accession numbers ranging from GCST0001391 to GCST0002121 [24] (Supplementary Table S1). This study utilized a comprehensive dataset containing 731 immune phenotypes, which were classified based on absolute cell counts (AC, *n* = 118), median fluorescence intensity reflecting surface antigen levels (MFI, *n* = 389), morphological parameters (MP, *n* = 32), and relative cell counts (RC, *n* = 192). The MFI, AC, and RC features included B cells, conventional dendritic cells (cDCs), mature T cells, monocytes, bone marrow cells, TBNK (T cells, B cells, NK cells), and Treg panel. The MP parameters (FSC-A and SSC-A) differentiated the size and intracellular complexity of immune cells for classification, including cDCs and TBNK panel (Appendix 1). This research conducted original GWAS studies on immune features using data from 3,757 European individuals, without overlapping cohorts. Approximately 22 million SNPs genotyped using high-density chips were analyzed for association using a reference panel based on the Sardinian sequence, adjusting for covariates such as gender and age [25].

### Selection of instrumental variables

In our study, we identified instrumental variables (IVs) based on three primary criteria. Firstly, we selected SNPs with a P-value of less than 1 × 10^–5^ from the GWAS data related to each immune trait [26, 27]. To ensure the independence of the chosen SNPs, we employed the PLINK tool (version v1.90) to filter out SNPs exhibiting linkage disequilibrium (LD) r2 values greater than 0.1 within a 500 kb range [28]. Following this step, we assessed the strength of the selected SNPs as instrumental variables by calculating the F-statistic for each immune trait. Generally. When the F-value surpasses 10, the SNP is deemed suitable for subsequent MR analysis [29]. Ultimately, we performed MR analysis on immune traits with more than two SNPs and ultimately included 729 immune cells.

### MR analysis

In this MR analysis, inverse variance weighted (IVW) method was primarily used to evaluate the causal relationship between immune traits and LUSC as well as LUAD. The IVW estimates are derived from a meta-analysis of the Wald ratios for all genetic variations [30]. IVW is based on the assumption that there is no horizontal pleiotropy across all SNPs, under which IVW provides the most accurate assessment of causal effects [31]. Therefore, we initially used IVW-based estimates to screen for immune cells that have causal impacts on LUSC and LUAD. The weighted median (WM) and MR-Egger methods were defined as the complementary analysis [32]. These two methods can provide more robust estimates under relaxed conditions. WM allows for less than 50% of SNPs to be ineffective [33], whereas MR-Egger provides detection of horizontal pleiotropy and heterogeneity when horizontal pleiotropy is present in all SNPs [30, 33]. However, inaccuracies may arise in the analysis outcomes when certain instrumental variables deviate from these assumptions. To tackle this problem, we conducted a series of sensitivity analyses. Initially, the Q-test method was utilized to evaluate potential heterogeneity among individual IVs, and p-value less than 0.05 from the Cochran Q test is considered indicative of heterogeneity in the results [34]. Subsequently, the MR-Egger intercept test was applied to estimate horizontal pleiotropy, guaranteeing that genetic variation has an independent relationship with both the exposure and outcome [35]. We used MR-PRESSO to re-examine the presence of heterogeneous SNPs [36]. Additionally, we conducted a leave-one-out (LOO) analysis, assessing whether the results were significantly influenced by individual SNPs by sequentially dropping each SNP and then performing MR analysis [33]. In summary, we rigorously screened for immune traits with potential causal effects on LUSC as well as LUAD through various criteria: (1) significant p-values in preliminary analysis (*p* < 0.05 from IVW) that remained significant after FDR correction; (2) Consistency in direction and magnitude across three MR methods; (3) MR results showed no heterogeneity or horizontal pleiotropy; (4) MR estimates were not severely disrupted by individual SNPs [28].

### Replication and meta-analysis

**Fig. 2:**
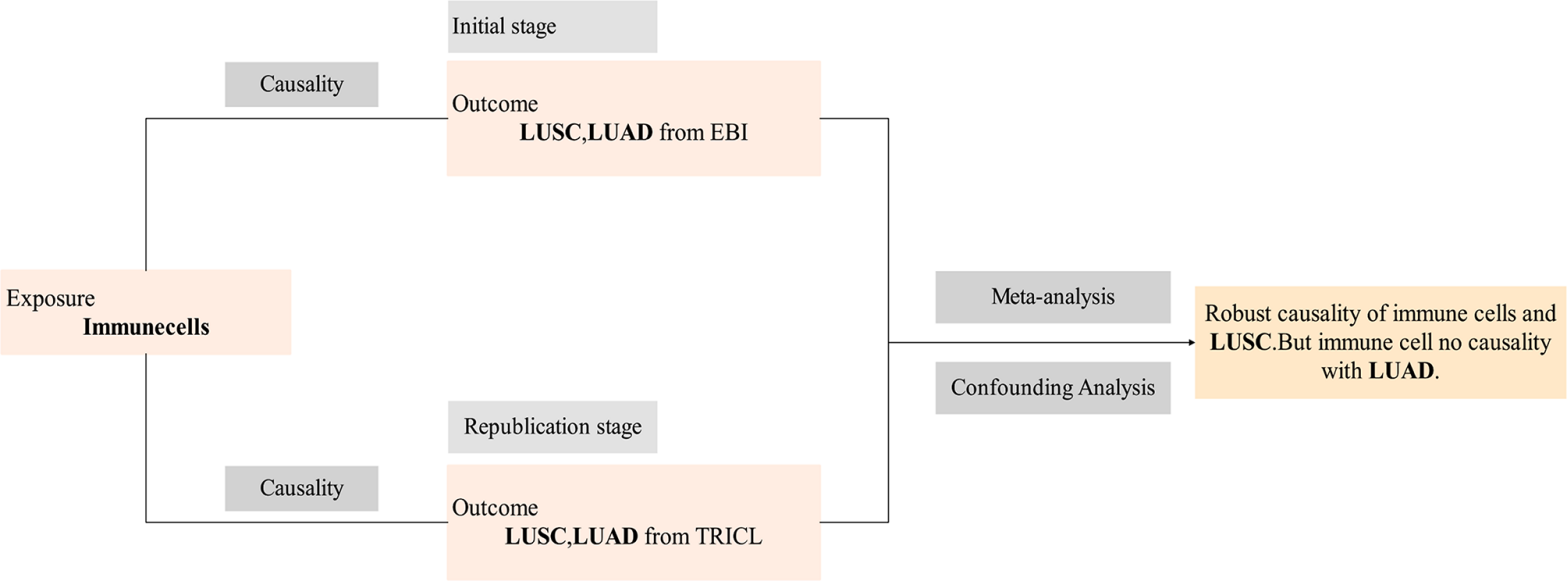
Technical roadmap of this study. *LUSC* lung squamous cell carcinoma; *LUAD* lung adenocarcinoma; *EBI* European Bioinformatics Institute; *TRICL* Transdisciplinary Research Into Cancer of the Lung

To comprehensively assess the stability of the candidate immune traits that were selected based on the criteria mentioned earlier, we conducted a replication of the IVW analysis in an alternative LUSC and LUAD cohort for validation [37]. This cohort is derived from the Transdisciplinary Research Into Cancer of the Lung (TRICL) study, including 18,946 European ancestry lung cancer cases and 109,382 European ancestry controls, with specific subgroups for LUSC (*N*_case_=7,704 and *N*_control_= 54,763) and LUAD (*N*_case_=11,245 and *N*_control_= 54,619). In this replication analysis, the GWAS data for lung cancer were sourced from the GWAS catalog, with accession numbers ieu-a-989 and ieu-a-984. To summarize, the initial analysis was conducted using GWAS data with accession numbers GCST004750 and GCST004744, while the replication analysis utilized GWAS data with accession numbers ieu-a-989 and ieu-a-984 (Fig. 2). Ultimately, we merged the outcomes of the two MR analyses through a meta-analysis to discern the immune traits with a causal effect on LUSC and LUAD (Supplementary Table S2). The meta-analysis was executed using the Generic Effect IVW model in Review Manager 5.4 software.

### Confounding analysis

In order to assess the potential horizontal pleiotropy of our MR results, we conducted several sensitivity tests to identify SNPs that may violate the MR assumptions. Nevertheless, it remains possible that some pleiotropic SNPs, which are difficult to detect, might still exist. To further evaluate whether each SNP is associated with established risk factors of LUSC and LUAD, including smoking [38], rheumatoid arthritis [39], body mass index [40], pollution [41] and genetic factors [42], we utilized the PhenoScanner V2 [43] website (http://www.phenoscanner.medschl.cam.ac.uk/) to scrutinize immune trait-related instrumental variables (IVs). If we identify any SNPs are significantly linked to the aforementioned confounding factors (p-value < 1 × 10^− 5^), we will exclude these SNPs and re-conduct the MR analysis. This crucial step aims to ensure the robustness and reliability of our analysis results.

### Statistical analysis

All statistical analyses were performed using R version 4.3.0. Specifically, for MR analysis, we employed the MendelianRandomization package 0.9.0 [44]. and TwoSampleMR package 0.5.7, and for MR-PRESSO analysis, we utilized the MRPRESSO package 1.0 [45]. In instances where multiple testing was involved, we applied the False Discovery Rate (FDR) method for correction, thereby effectively mitigating the risk of false-positive findings. A statistically significant association with lung cancer was deemed present when the FDR value for the estimated causal effect of a particular immune trait was less than 0.05.

### Data availability

All data used in this study are publicly available. No human subject approvals were necessary to conduct these analyses. All the data can be found from the GWAS directory (https://gwas.mrcieu.ac). The serial numbers of the immune cells are from GCST0001391 to GCST0002121, respectively. For the LUSC and LUAD data used for the primary analysis, the serial number is GCST004750 and GCST004744, and the LUSC and LUAD serial numbers used for the replication and meta-analysis are ieu-a-989 and ieu-a-984.

## Results

### Selection of IVs

For LUSC, the number of IVs for the 729 selected immune phenotypes ranged from 3 to 1182, with a median of 27. The minimum F-statistic values for validity testing consistently exceeded 10, with a range of 20 to 63 (Supplementary Table S3). Similarly, for LUAD, the number of IVs ranged from 3 to 1196. The minimum F-statistic values for their validity testing surpassed 10, spanning from 20 to 60 (Supplementary Table S4). These results indicate that the potential bias from weak instruments has been adequately resolved.

### Preliminary analysis of lung cancer risk on immunophenotypes

**Fig. 3:**
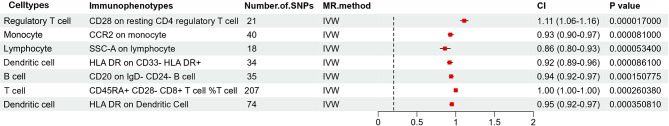
Forest plots showed the causal associations between lung squamous cell carcinoma and immune cell traits by IVW. *IVW* inverse variance weighting; *CI* confidence interval; *SNP* single nucleotide polymorphisms

To assess the causal effects of immunophenotypes on LUSC or LUAD, our primary analytical approach was the IVW method. Utilizing the FDR method for multiple tests, we identified seven suggestive immunophenotypes with a significance level of 0.05 in LUSC (Fig. 3). In contrast, no suggestive immunophenotypes were detected at this significance level in LUAD (Supplementary Table S5).

Consistency was then observed in the direction and magnitude of the IVW, MR-Egger, and WM estimates across these seven immune cells (Supplementary Fig. S1). Our subsequent analysis involved a further evaluation of these seven immune cell traits. We excluded four immune cells - SSC-A on lymphocyte, HLA DR on CD33- HLA, CD20 on IgD- CD24- B cell, and HLA DR on Dendritic Cell - based on not meeting the criteria of Q-test method *p* < 0.05, or MR-Egger intercept test *p* < 0.05, or MR-PRESSO method *p* < 0.05(Supplementary Table S5).

The refined analysis focused on the remained three types of immune cells, comprising two from the Treg panel and one from the cDC panel. Our analysis revealed two subtypes associated with increased risks of LUSC: CD28 on resting CD4 regulatory T cells (OR = 1.11, 95% CI = 1.06–1.16, *p* = 1.70E-05, FDR = 1.2E-02) and CD45RA + CD28- CD8 + T cell %T cell (OR = 1.00, 95% CI = 1.00–1.00, *p* = 2.60E-04, FDR = 3.10E-02). Additionally, we identified an immunophenotype, CCR2 on monocytes (OR = 0.93, 95% CI: 0.90–0.97, *p* = 8.10E-5, FDR = 1.6E-02), exhibiting protective effects against LUSC susceptibility.To address potential biases in MR estimation for these three immune cell types, we conducted a leave-one-out (LOO) analysis, confirming that no single SNP caused significant bias.

**Fig. 4:**
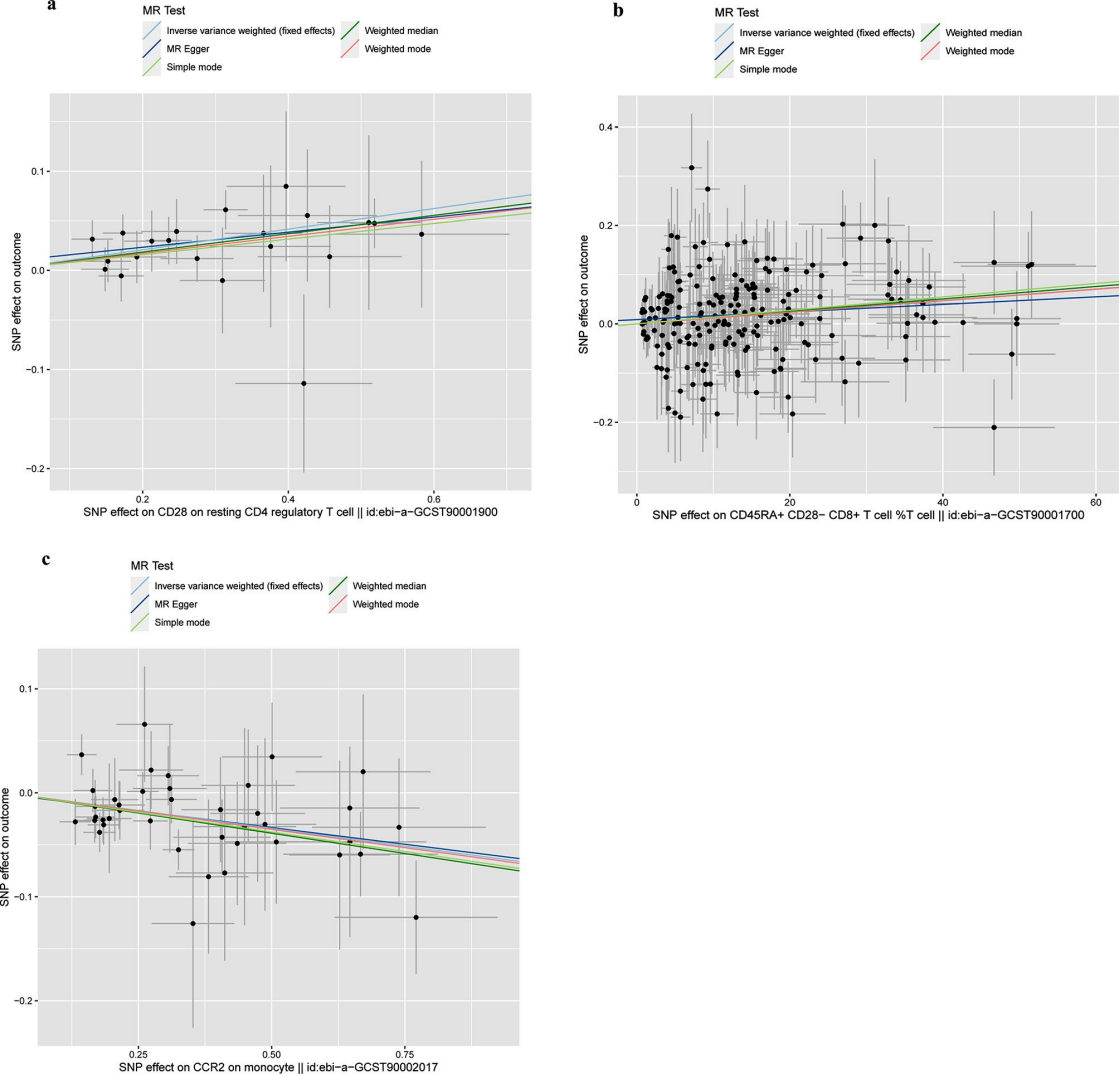
Causal effects of lung squamous cell carcinoma on immune cells concentration. *SNP* single nucleotide polymorphisms. **a**: Scatter plot between CD28 on resting CD4 regulatory T cells and LUSC risk. **b**: Scatter plot between CD45RA + CD28- CD8 + T cell %T cell and LUSC risk. **c**: Scatter plot between CCR2 on monocytes and LUSC risk

In summury, The IVW estimates for the three selected immune cells were significant, maintained significance after FDR correction, and were consistent in direction and magnitude (Fig. 4). The Cochran Q Test (*p* > 0.05) and MR-Egger intercept test (*p* > 0.05) indicated no heterogeneity or pleiotropy for these immune cells. Similarly, MR-PRESSO results, after outlier removal, suggest the absence of heterogeneous SNPs (Table 1). The LOO analysis further supported the reliability of our MR estimation, as shown in Supplementary Figure S1. These findings led us to consider these three immune cells as prime candidates for further analysis (Supplementary Fig. S2).

**Table 1:** Causal Relationship Between Positive immunophenotypes Identified by IVW Method and lung Squamous Cell Carcinoma

immune cell	Number of SNPs	Beta	OR (95%CI)	*P*	*P* for heterogeneity test	*P* for MR-Egger intercept	*P* for MR-PRESSO (0 outliers)
**CD28 on resting CD4 regulatory T cell**							
IVW	21	0.104	1.11 (1.06–1.16)	1.70E-5	0.946	0.596	0.943
MR Egger	21	0.076	1.08 (0.96–1.21)	2.00E-1			
Weighted median	21	0.092	1.10 (1.02–1.18)	1.10E-2			
**CD45RA + CD28- CD8 + T cell %T cell**							
IVW	207	0.001	1.00 (1.00–1.00)	2.60E-4	0.425	0.134	0.439
MR Egger	207	0.0007	1.00 (1.00–1.00)	7.10E-2			
Weighted median	207	0.001	1.00 (1.00–1.00)	1.20E-2			
**CCR2 on monocyte**							
IVW	40	-0.068	0.93 (0.90–0.97)	8.10E-5	0.904	0.903	0.925
MR Egger	40	-0.064	0.94 (0.87–1.01)	9.90E-2			
Weighted median	40	-0.078	0.93 (0.88–0.97)	2.00E-3			

**IVW** inverse variance weigh

### Replication and meta-analysis

To enhance the persuasiveness of our estimates, we conducted a replication of the MR analysis using additional GWAS data related to LUSC and LUAD. Similarly, we also found same three suggestive immunophenotypes at a significance level of 0.05 (Supplementary Table S6), while we did not identify any suggestive immunophenotypes with a significance level of 0.05 in LUAD (Supplementary Table S7). As anticipated, we observed similar trends in candidate immunophenotypes in this new LUSC dataset. It’s important to note that, despite these consistent trends, the results did not achieve P FDR < 0.05 statistical significance in replication analysis. This lack of significance can be attributed primarily to the minor differences in experimental conditions or study designs.

**Fig. 5:**
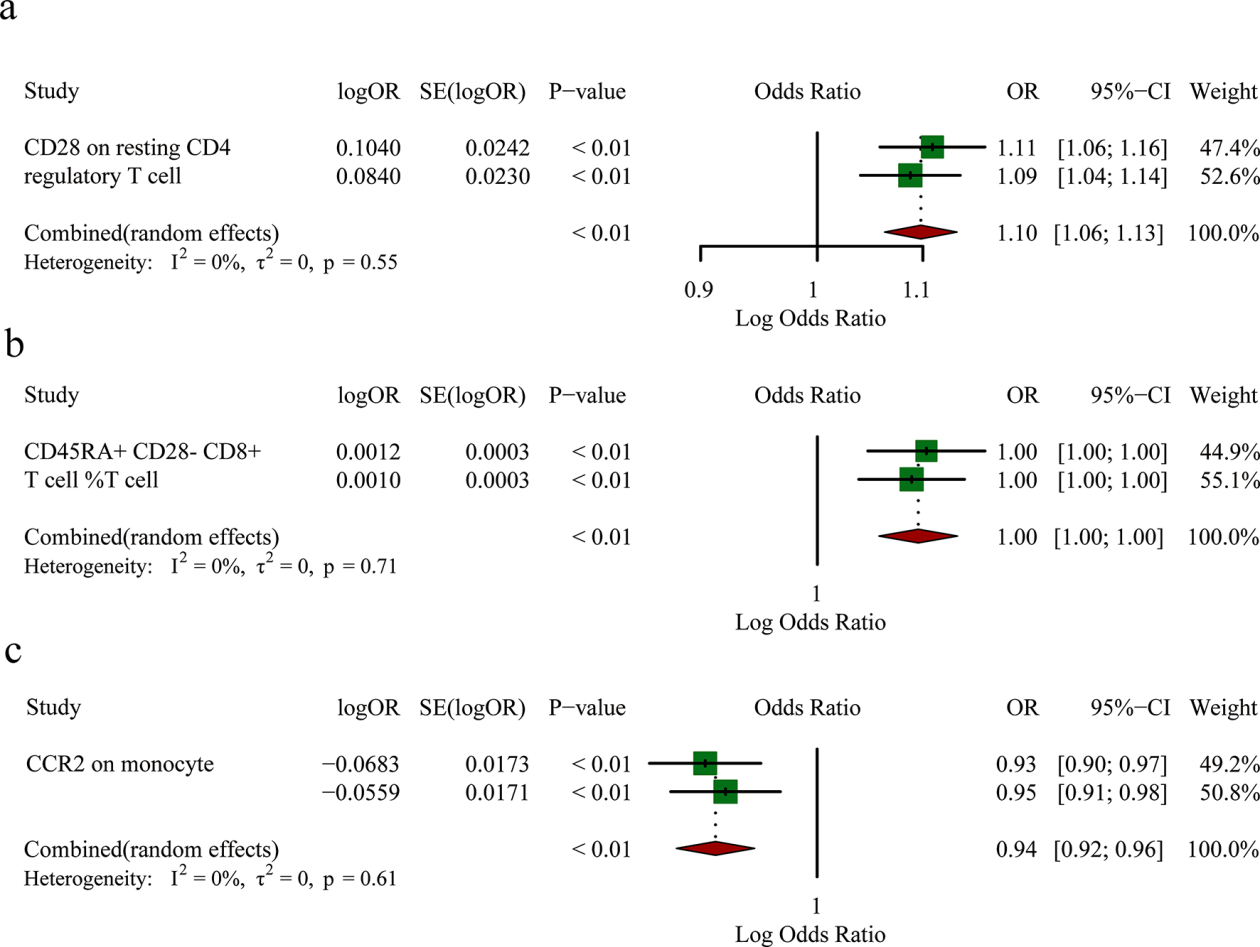
Meta-analysis of significantly associated (IVW derived *p* < 0.05) between immune cells and lung Squamous Cell Carcinoma. 95% CI, 95% confidence interval; OR, odds ratio. **a**: Meta plot between CD28 on resting CD4 regulatory T cells and LUSC. **b**: Meta plot between CD45RA + CD28- CD8 + T cell %T cell and LUSC. **c**: Meta plot between CCR2 on monocytes and LUSC

To strengthen our findings, we combined the outcomes of the GWAS data and conducted a meta-analysis that yielded further insights. As shown in Supplement Table S8, the analysis validated the causal relationship between three specific immunophenotypes and LUSC: CD28 expression on resting CD4 regulatory T cells (OR 1.10, 95% CI: 1.06–1.13, *p* < 0.01) and CD45RA + CD28- CD8 + T cell %T cell (OR 1.00, 95% CI: 1.00–1.00, *p* < 0.01), suggesting increased risk of LUSC; the expression of CCR2 on monocytes (OR 0.94, 95% CI: 0.92–0.96, *p* < 0.01) was associated with a reduced risk of LUSC (Fig. 5). Notably, these findings exhibited consistent directions in both MR analyses, and the meta-analysis yielded statistically significant estimations (Supplementary Table S8).

### Confounding analysis

To ensure that our instrumental variables (IVs) are independent of confounding factors, we examined our selected SNPs for their autonomy from common risk factors for LUSC. Specifically, we assessed their associations with recognized risk factors such as smoking, RA, BMI, pollution and genetic predispositions. In the CD45RA + CD28-CD8 + T cell percentage, three SNPs were identified as being associated with the risk factors for LUSC (Supplementary Table S9). The remaining immune cells did not identify the suspected SNP. Re-analysis with exclusion of three SNPs demonstrated that our estimates remain significant: CD45RA + CD28- CD8 + T cell percentage: (OR 1.00 95% CI: 1.00–1.00, *p* < 0.01).

## Discussion

In this study, we integrated two extensive GWAS datasets to investigate the causal relationships between 731 genetically proxied immune cell traits and LUSC or LUAD through a Mendelian randomization design. Our study suggests that CD28 on resting CD4 regulatory T cells and CD45RA + CD28- CD8 + T cell %T cell increases the risk of LUSC, while a phenotype of CCR2 on monocytes is associated with a reduced risk of LUSC. However, we did not find any immune cells with a causal relationship in LUAD. Our conclusions were further reinforced by replication and meta-analysis, and subsequently, our estimates remained significant after excluding SNPs associated with confounding factors. To our knowledges, this study represents the first MR investigation to utilize comprehensive GWAS data on immune cell traits to explore their causal links with LUSC and LUAD.

Our study revealed that the expression of CD28 on resting CD4 regulatory T cells correlates with an increased risk of LUSC. CD28 is a transmembrane protein located on the surface of T cells, which is crucial for signal transduction during T cell activation [46]. Experimental studies indicate that CD28 conditional knockout results in severe autoimmunity and inappropriate resolution of allergy, suggesting that CD28 plays post-maturational roles in Tregs [47]. The expression of CD28 on CD4 + Treg is essential for Treg homeostasis, and its interaction with B7 is required for their immunosuppressive function [48, 49]. This phenotype suggests an immunosuppressive milieu that may raise the vulnerability of LUSC, corroborating our MR analysis findings.

Meanwhile, the study found that the CD45RA + CD28- CD8 + T cell correlated with increased risk of LUSC. According to Orrù et al., we categorized CD8 + T cells into naïve T cells (CD45RA + CCR7+), central memory T cells (CD45RA − CCR7+), effector memory T cells (CD45RA − CCR7−), and terminally differentiated T cells (CD45RA + CCR7−) [24]. There are reports suggesting that the CD45RA + CD28 − CD8 + T cell subset appears to be similar to terminally differentiated CD45RA + CD8 + T cells with negative staining for the chemokine receptor CCR7 [50–54]. Terminally differentiated T cells exhibit lower proliferative capacity, reduced differentiation plasticity, but have strong effector activity (such as cytotoxicity and cytokine release), even without T cell receptor cross-linking through antigen exposure (via bystander activation through cytokine receptor) [55]. Terminally differentiated T cells are frequently linked to chronic inflammatory conditions in the setting of aging and chronic infections [56, 57]. Recent studies have confirmed that the terminally differentiated T cells and its CD28- subsets have the potential to serve as a biomarker of immunosenescence in the context of cytomegalovirus infection [58]. The absence of CD28 expression on CD8 + TEMRA possibly indicates weak T cell receptor engagement, suggesting weaker protective effects against infection and cancer development. These findings are in harmony with our MR analysis, indicating that this phenotype was associated with an increased risk of LUSC. Additionally, research has found that terminally differentiated CD8 + T cells originate from TCF-1 + stem-like T cells in the tumor microenvironment [59]. Activation of STAT3 signaling by IL-10 and IL-21 promotes the development and survival of terminally differentiated T cells [60]. Activated terminally differentiated T cells are producers of inflammatory cytokines, and the inflammatory cytokines found in tumors are more likely to promote tumor growth, progression, and immune suppression rather than eliciting effective host anti-tumor responses [61, 62]. Therefore, further research and analysis are needed to elucidate the exact role of CD45RA + CD28- CD8 + T cells in LUSC.

Notably, our study revealed one immune cell trait, CCR2 on monocytes, as a protective factor against LUSC. CCR2 is generally considered to have a detrimental role in various cancers, particularly in prostate cancer [63]. But in our MR analysis, the expression of CCR2 on monocytes seems to be a protective factor in LUSC. This finding challenges the existing research on the function of CCR2 in cancer.

The role of CCR2 in cancer is complex and diverse. CCR2 is primarily expressed by monocytes/macrophages with strong pro-inflammatory functions [63], which is expected to prevent carcinogenesis. Paradoxically, increased expression of CCL2, a molecule that interacts with CCR2, has been linked to the accumulation of tumor-associated macrophages in esophageal squamous cell carcinoma (ESCC). These macrophages exhibit potent immunosuppressive activities within the tumor microenvironment, which could potentially facilitate cancer progression [64, 65].

Moreover, it has been observed that CCR2 can play a dual role in cancer. On one hand, it may boost immune responses against tumors by encouraging the migration of immune cells to the tumor site. On the other hand, once these CCR2-positive immune cells are within the tumor microenvironment, they might contribute to tumor growth, metastasis, and immune evasion through their interaction with CCL2 [66, 67]. This dual role presents a paradox: while CCR2 is expected to aid in cancer prevention through its pro-inflammatory action, it can also inadvertently support tumor progression in certain contexts. Our MR analysis, which suggests a protective role of CCR2 in LUSC, adds to this complexity. These conflicting perspectives highlight the need for more in-depth investigations to fully understand the multifaceted role of CCR2 in cancer biology.

These findings suggest a dualistic and context-dependent role of CCR2 in cancer, which our study contributes to by highlighting its potential protective role in LUSC. This underscores the need for a more detailed understanding of the diverse functions of immune cell traits in cancer, particularly in lung squamous cell carcinoma. Our research adds to the growing body of evidence that the role of immune cells in cancer is not straightforward and warrants further investigation.

Cancer cells exhibit less invasiveness in never-smoking LUAD, while the immune environment shows more immunosuppressive effects, indicating vulnerability in the treatment of LUAD [68]. Studies have found that smoking-induced dysfunction of alveolar cells contributes more to the aggressiveness of LUAD in smokers, while the immunosuppressive microenvironment has a greater impact on the aggressiveness of LUAD in never-smokers [69]. Never-smoking LUAD patients have unique subtypes of cancer cells expressing high levels of MHC-II molecules involved in antigen presentation and activation of anti-tumor immunity [70]. However, we did not find a relationship between immune cell characteristics and LUAD in our study, possibly because of the close association between LUAD and smoking, which we did not differentiate between smokers and non-smokers. Additionally, pulmonary involvement is a common extra-articular manifestation of RA [71]. Immune dysregulation is considered a key factor in RA patients developing LUAD. Bioinformatics studies have shown that CD8A, GZMA, and PRF1 are associated with CD8 T cells in RA and positively correlated with 33 types of tumors [72]. Shi et al. also found common physiological and pathological processes and molecular spectra between RA and LUAD [73]. Therefore, we finally excluded confounding factor-related SNPs, and the obtained SNPs were only related to immune cells, thereby influencing lung cancer.

This study also has some limitations. Firstly, MR analysis cannot replace clinical trials in the objective field, as it is only a method of analyzing causality between exposure and outcome. Therefore, further research is needed to confirm the potential association between immune cells and lung cancer risk. Secondly, this study is based on publicly available GWAS data, and our MR analysis was conducted only in European populations. Given the genetic specificity among different races, it may not be applicable to other populations. Future studies should conduct subgroup analysis including different populations to draw more comprehensive conclusions. Thirdly, our observations on CCR2 + monocytes differ from current literature viewpoints. Such discrepancies may arise due to variations in sample sources, experimental conditions, or statistical methods. Biological complexity in dynamic systems of the tumor microenvironment may also be a relevant factor. Thus, investigating the precise role of circulating CCR2 + monocytes in LUSC risk becomes necessary, considering the current controversy. Finally, our study primarily examines specific immune cell traits, but we cannot disregard the impacts of other cell types or factors that may impact the risk of LUSC. For instance, certain tumor-infiltrating myeloid cell subsets, m6A regulation, genomic alterations, and specific somatic mutations can play pivotal roles in regulating the interactions between various immune cells and influence tumor development [74–77]. Therefore, while laying the foundation in our study, it is also necessary to gain a more comprehensive understanding of these factors and their interactions. We will strive to improve in future research endeavors.

In summary, we performed a two-sample Mendelian randomization study to investigate the causal connections between different immune phenotypes and LUSC or LUAD. Our analysis demonstrated that the causal relationship is more pronounced in LUSC and three immune cell types correlated with LUSC susceptibility, while the association with LUAD is statistically insignificant. Although these findings are derived only from European populations, they still enhance our comprehension of the complex interplay between the immune system and lung cancers. This study yields new perspectives into managing cancer risk, potentially providing more precise therapeutic alternatives for individuals with lung cancer and furnishing valuable information for future scientific research.

### Electronic supplementary material

Below is the link to the electronic supplementary material.

Supplementary Material 1

Supplementary Material 2

Supplementary Material 3

Supplementary Material 4

Supplementary Material 5

Supplementary Material 6

## Data Availability

The GWAS datasets analyzed during this study are publicly accessible. The initial analysis was conducted using GWAS data with accession numbers GCST004750 and GCST004744, available from the GWAS Catalog. The replication analysis utilized GWAS data with accession numbers ieu-a-989 and ieu-a-984, which can be accessed through the IEU OpenGWAS project.
