# Supplementary figures and images for: Associations between immune cell phenotypes and lung cancer subtypes: insights from mendelian randomization analysis
Source: BMC Pulm Med. 2024 May 16;24:242. doi: 10.1186/s12890-024-03059-w (PMC11100125; doi:10.1186/s12890-024-03059-w)

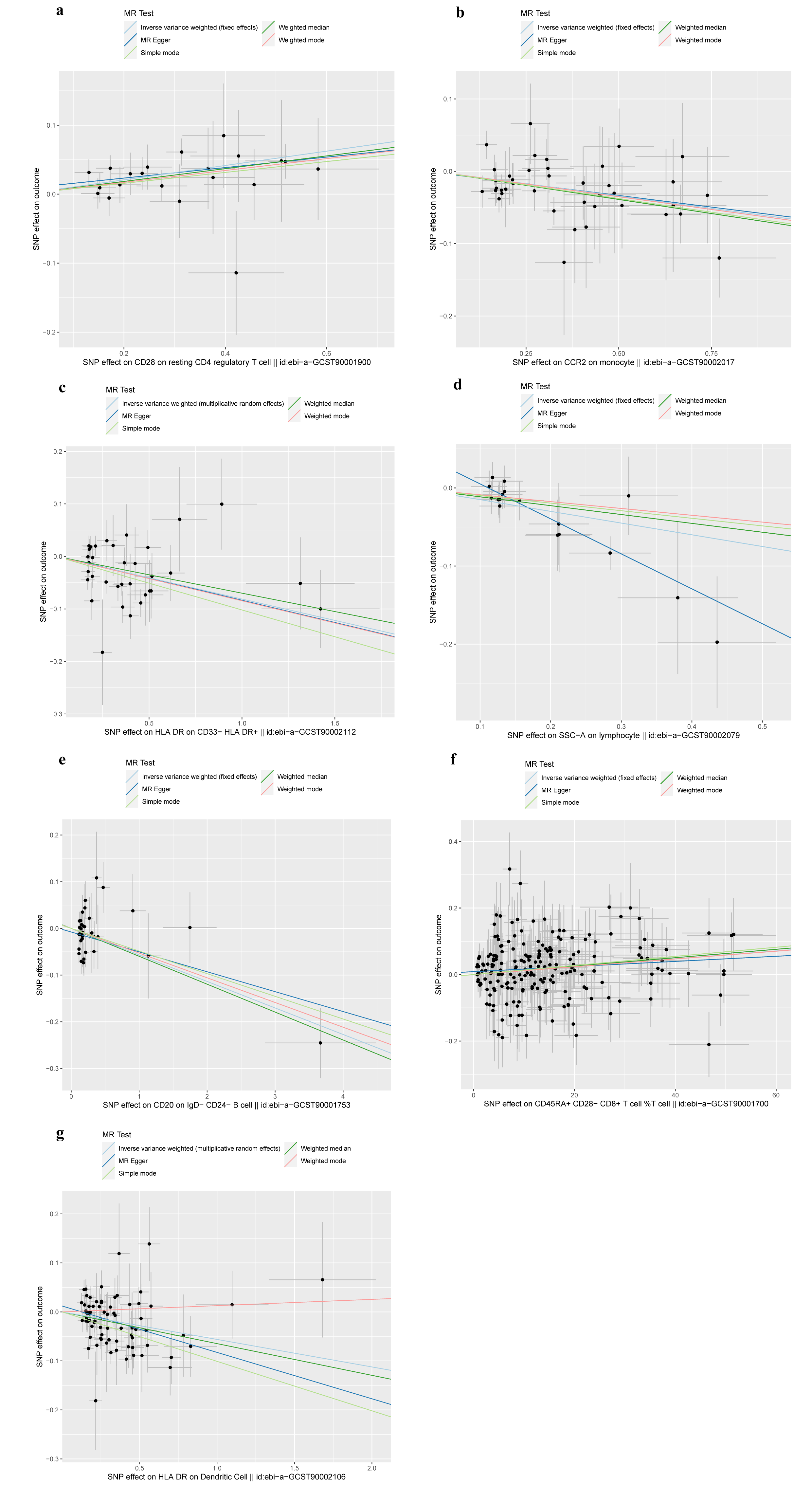

Supplement: Supplementary file 1 — Supplementary Material 1 [file 12890_2024_3059_MOESM1_ESM.tif]

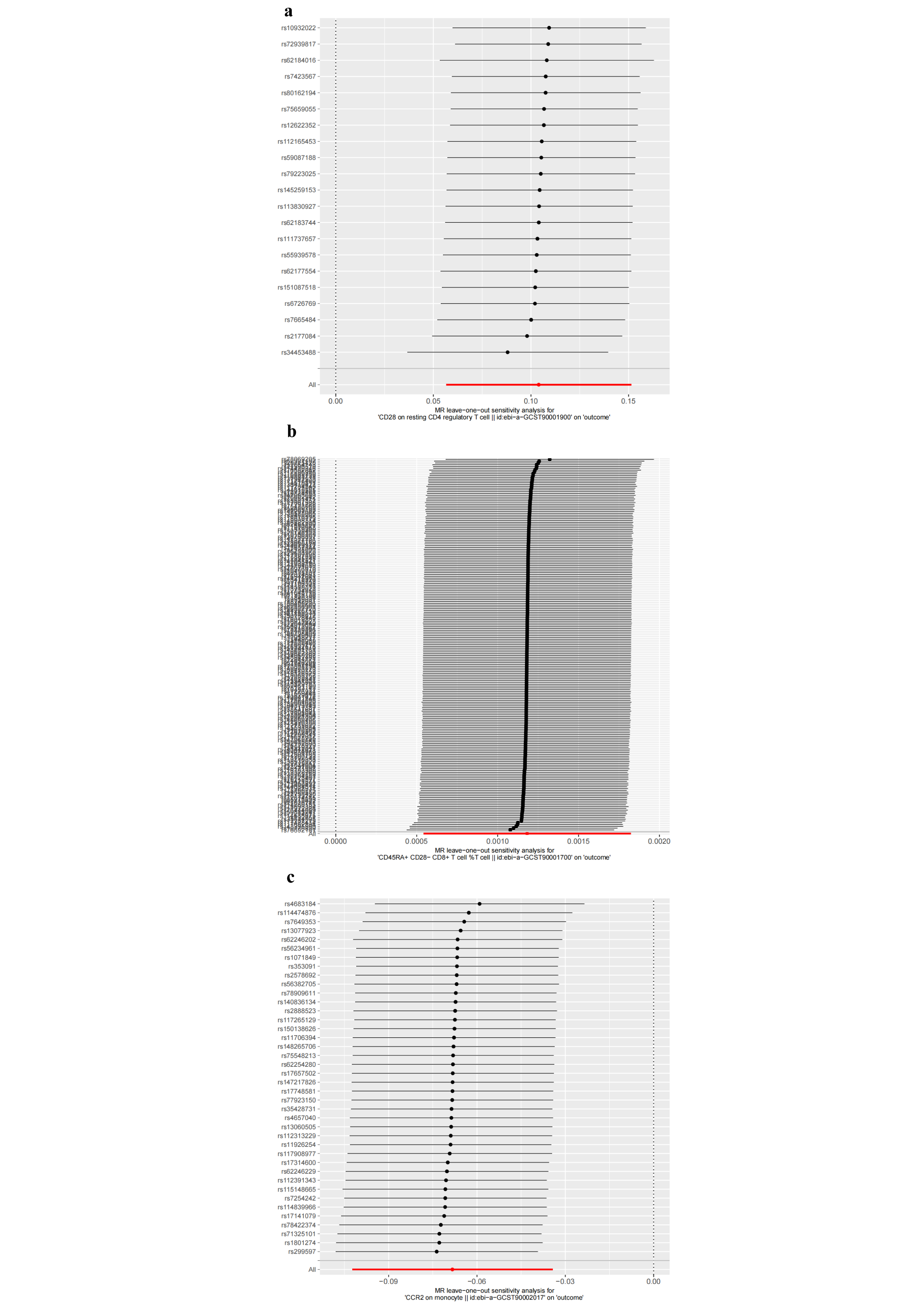

Supplement: Supplementary file 2 — Supplementary Material 2 [file 12890_2024_3059_MOESM2_ESM.tif]
