# Supplementary material for: Associations between immune cell phenotypes and lung cancer subtypes: insights from mendelian randomization analysis
Source: BMC Pulm Med. 2024 May 16;24:242. doi: 10.1186/s12890-024-03059-w (PMC11100125; doi:10.1186/s12890-024-03059-w)
Supplement: Supplementary file 3 — Supplementary Material 3 [file 12890_2024_3059_MOESM3_ESM.docx]

**Supplementary Materials**

**Associations between Immune Cell Phenotypes and Lung Cancer Subtypes: Insights from Mendelian Randomization Analysis**

Jin-Min Zheng^1^, Chen-Xi Lou^2^, Yu-Liang Huang^1^, Wen-Tao Song^3^, Yi-Chen Luo^4^, Guan-Yong Mo^5^, Lin-Yuan Tan^1^ and Shang-Wei Chen^4#^, Bai-Jun Li^6#^

^1^ Department of Surgery, Guangxi Medical University, Nanning, Guangxi, China.

^2^ Department of Surgery, Guangxi University of Chinese Medicine, Nanning, Guangxi, China.

^3^ Department of Surgery, Youjiang Medical University For Nationalities, Baise, Guangxi, China.

^4^ Department of thoracic surgery, Guangxi Academy of Medical Sciences and the People's Hospital of Guangxi Zhuang Autonomous Region, Nanning, Guangxi, China.

^5^ Department of thoracic surgery, Guilin Medical University, Guilin, Guangxi, China.

^6^ Department of thoracic surgery, Tumor Hospital of Guangxi Medical University, Nanning, Guangxi, China.

These authors contributed equally: Jin-Min Zheng and Chen-Xi Lou

#Corresponding Author: Shang-Wei Chen and Bai-Jun Li

**Supplemental Fig S1-S2**

**Supplemental Table S1-S9** (in excel file)

**
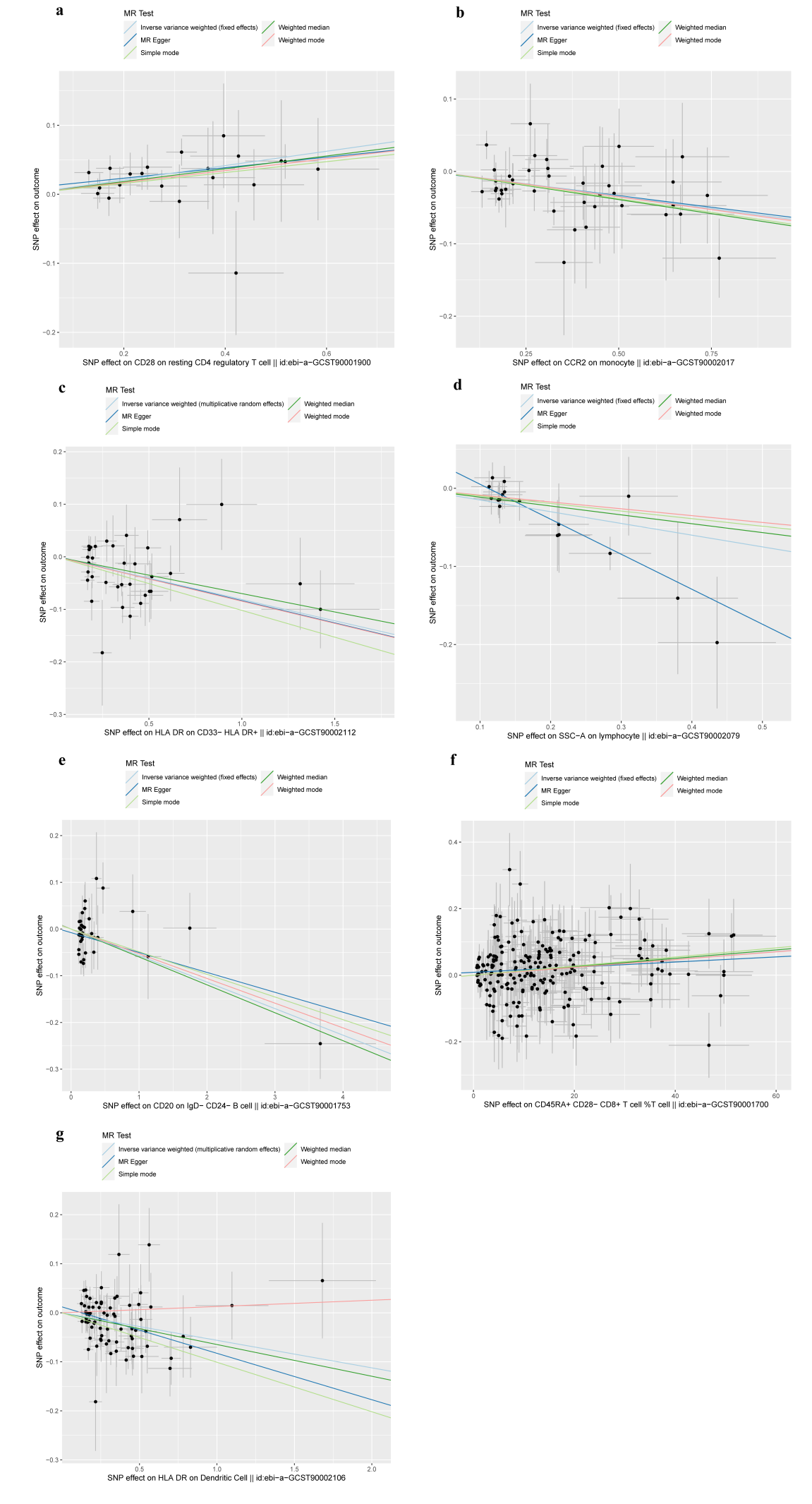
**

**Supplementary Fig. S1**. Causal effects of lung Squamous Cell Carcinoma on immune cells concentration. SNP, single nucleotide polymorphisms.

a: MR leave-one-out plot between CD28 on resting CD4 regulatory T cell and LUSC;

b: MR leave-one-out plot between CCR2 on monocyte and LUSC;

c: MR leave-one-out plot between SSC-A on lymphocyte and LUSC;

d: MR leave-one-out plot between HLA DR on CD33- HLA DR+ and LUSC;

e: MR leave-one-out plot between CD20 on IgD- CD24- B cell and LUSC;

f: MR leave-one-out plot between CD45RA+ CD28- CD8+ T cell %T cell and LUSC;

g: MR leave-one-out plot between HLA DR on Dendritic Cell and LUSC;

**
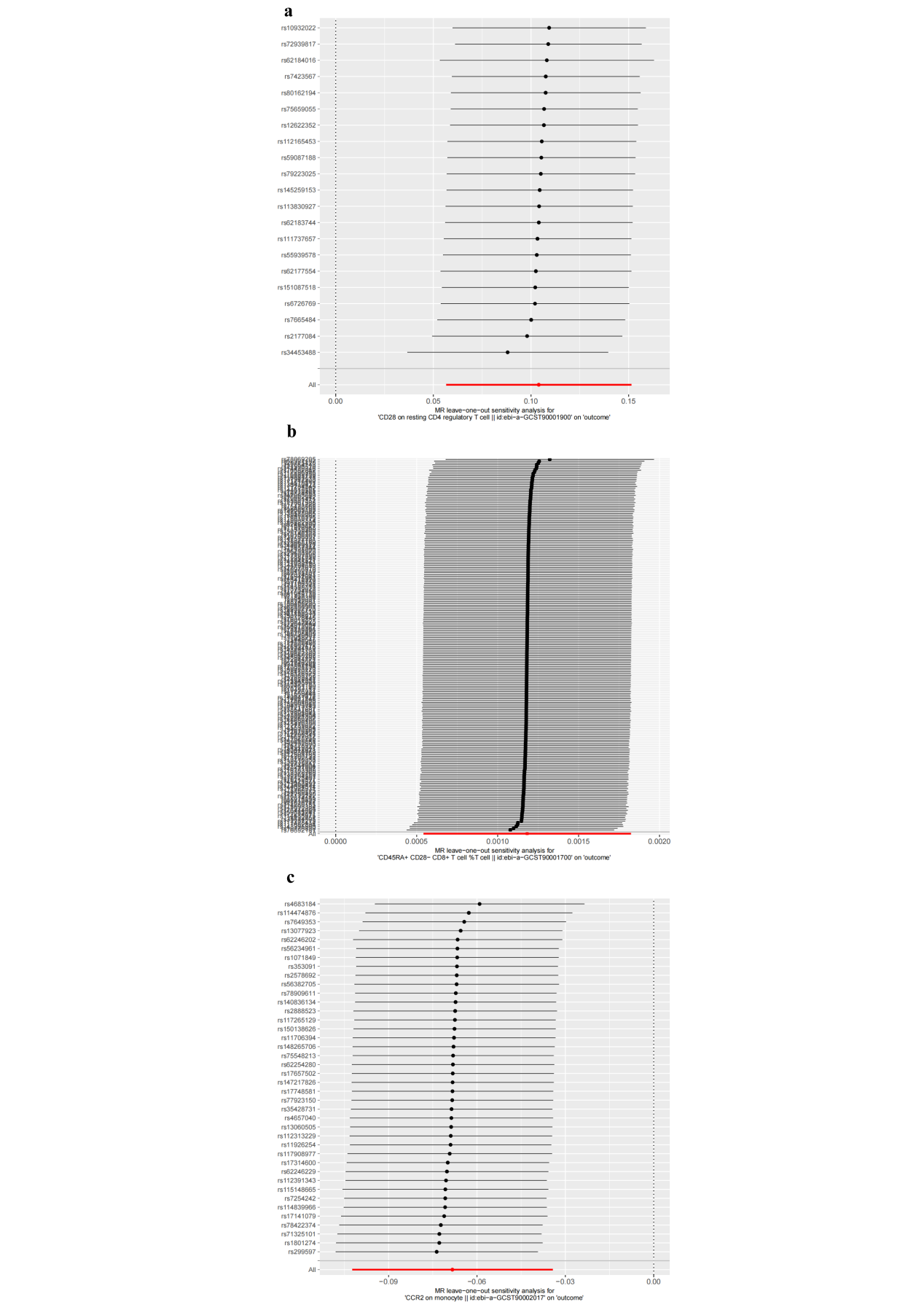
**

**Supplementary Fig. S2**. Forest plots for the Mendelian randomization (MR) leave-one-out analysis of the significant inverse variance weighted (IVW) estimates. a: MR leave-one-out plot between CD28 on resting CD4 regulatory T cells and LUSC;

b: MR leave-one-out plot between CD45RA+ CD28- CD8+ T cell %T cell and LUSC; c: MR leave-one-out plot between CCR2 on monocytes and LUSC.
