# Supplementary material for: Associations between immune cell phenotypes and lung cancer subtypes: insights from mendelian randomization analysis
Source: BMC Pulm Med. 2024 May 16;24:242. doi: 10.1186/s12890-024-03059-w (PMC11100125; doi:10.1186/s12890-024-03059-w)
Supplement: Supplementary file 4 — Supplementary Material 4 [file 12890_2024_3059_MOESM4_ESM.docx]

**Associations between Immune Cell Phenotypes and Lung Cancer Subtypes: Insights from Mendelian Randomization Analysis**

#Corresponding Author:

Shang-Wei Chen

Department of thoracic surgery, Guangxi Academy of Medical Sciences and the People's Hospital of Guangxi Zhuang Autonomous Region

Phone: 0086-0771-2186755;

Email: ShangweiChen_gxmu@126.com

Bai-Jun Li

Department of thoracic surgery, Tumor Hospital of Guangxi Medical University

Phone: 0086-0771-2186755;

Email: libaijun1979@126.com

**STROBE-MR checklist of recommended items to address in reports of Mendelian randomization studies**^1^ ^2^

| **Item No.** | **Section** | **Checklist item** | **Page No.** | **Relevant text from manuscript** |
| --- | --- | --- | --- | --- |
| 1 | **TITLE and ABSTRACT** | Indicate Mendelian randomization (MR) as the study’s design in the title and/or the abstract if that is a main purpose of the study | No.1-2 | Title：Associations between Immune Cell Phenotypes and Lung Cancer Subtypes: Insights from Mendelian Randomization Analysis  Abstract:  Introduction:  Lung cancer's association with the immune environment is not fully understood. This study employs Mendelian Randomization (MR) to explore the causal relationship between immune cells and lung cancer subtypes, including lung squamous cell carcinoma (LUSC) and lung adenocarcinoma (LUAD).  Methods:  We analyzed the association between 731 immune cell phenotypes and LUSC or LUAD, sourced from the European Bioinformatics Institute (EBI). Sensitivity analyses, including MR-Egger intercept, Cochran Q test, and others, were conducted for robustness. The findings were cross-validated using Transdisciplinary Research Into Cancer of the Lung (TRICL) data. Confounding analysis was used to further ensure robustness of the results.  Results:  Among 731 immune cell phenotypes examined, three immune phenotypes exhibited statistically significant effects with LUSC. CD28 expression on resting CD4 regulatory T cells (OR 1.0980, 95% CI: 1.0627-1.1344, p < 0.0001) and CD45RA+ CD28- CD8+ T cell %T cell (OR 1.0011, 95% CI: 1.0007; 1.0015, p < 0.0001) were associated with increased susceptibility to LUSC. Conversely, CCR2 expression on monocytes (OR 0.9399, 95% CI: 0.9177-0.9625, p < 0.0001) was correlated with a decreased risk of LUSC. However, no significant causal relationship was established between any immune cell phenotypes and LUAD.  Conclusion:  The study identifies three immune cell phenotypes as potential risk factors for LUSC, but none for LUAD. Further experimental investigations are warranted for validation. |
|  | **INTRODUCTION** |  |  |  |
| 2 | **Background** | Explain the scientific background and rationale for the reported study. What is the exposure? Is a potential causal relationship between exposure and outcome plausible? Justify why MR is a helpful method to address the study question |  | Scientific Background and Rationale:  Context: This study focuses on lung cancer, specifically LUSC and LUAD, which are the second most common types of cancer globally. The pathogenesis of lung cancer is complex, involving genetic, environmental factors, and the role of the immune system.  Immune Cells and Lung Cancer: In recent years, the role of immune cells in the onset and progression of lung cancer has garnered increasing attention. Existing research suggests that immune cells, particularly regulatory T cells and monocytes, may play a significant role in the pathogenesis of lung cancer.  Exposure and Potential Causal Relationship:  Exposure: The exposure factor is 731 different immune cell phenotypes.  Causal Relationship Plausibility: The potential causal relationship between immune cells and lung cancer is plausible, as the immune system plays a key role in the onset, progression, and prognosis of cancer. Specifically, different phenotypes of immune cells may influence the susceptibility and progression of lung cancer.  Justification for Using MR:  Addressing Confounding and Reverse Causation: Traditional observational studies exploring the relationship between immune cells and lung cancer may be affected by confounding factors and reverse causation. The MR method, using genetic variants as instrumental variables, helps overcome these issues, providing more reliable causal inference.  Genetic Instrumentation: In this study, SNPs directly associated with immune cell phenotypes were chosen as instrumental variables, which are less likely to be influenced by confounding factors related to lung cancer risk.  Robustness to Confounding: A major advantage of the MR method is its robustness against traditional confounding factors, making it a powerful tool for exploring the relationship between immune cells and lung cancer. |
| 3 | **Objectives** | State specific objectives clearly, including pre-specified causal hypotheses (if any). State that MR is a method that, under specific assumptions, intends to estimate causal effects |  | Specific Objectives:  Primary Objective: To investigate the causal relationship between various immune cell phenotypes and both major subtypes of lung cancer: lung squamous cell carcinoma (LUSC) and lung adenocarcinoma (LUAD).  Secondary Objective: To identify specific immune cell phenotypes that may contribute to the risk or protection against LUSC and LUAD, thereby enhancing the understanding of the immunological underpinnings in both subtypes of lung cancer.  Pre-specified Causal Hypotheses:  Hypothesis 1: Certain immune cell phenotypes are causally associated with an increased risk of developing LUSC and potentially LUAD.  Hypothesis 2: Other immune cell phenotypes may provide a protective effect against the development of LUSC and LUAD.  Hypothesis 3: The causal relationships between immune cell phenotypes and LUSC and LUAD may vary, reflecting the distinct pathological characteristics of these subtypes.  Explanation of MR as a Method:  Method Description: This study employs Mendelian Randomization (MR), using genetic variants as instrumental variables to estimate causal effects between exposures (immune cell phenotypes) and outcomes (lung cancer subtypes LUSC and LUAD).  Underlying Assumptions: MR relies on the assumptions that genetic variants are associated with the exposure, not associated with confounders, and influence the outcome only through the exposure. Under these assumptions, MR aims to provide causal effect estimates, minimizing biases inherent in observational studies. |
|  | **METHODS** |  |  |  |
| 4 | **Study design and data sources** | Present key elements of the study design early in the article. Consider including a table listing sources of data for all phases of the study. For each data source contributing to the analysis, describe the following: |  |  |
|  | a) | Setting: Describe the study design and the underlying population, if possible. Describe the setting, locations, and relevant dates, including periods of recruitment, exposure, follow-up, and data collection, when available. |  | Design Overview: This study employs a two-sample Mendelian Randomization approach to explore the causal relationship between immune cell phenotypes and lung cancer subtypes, specifically LUSC and LUAD.  Population Basis: The analysis utilizes genetic data from large-scale genome-wide association studies (GWAS), representing a broad European population.  Data Sources:  GWAS Data for Lung Cancer: GWAS summary data for lung cancer, including LUSC and LUAD, were obtained from the GWAS Catalog (https://www.ebi.ac.uk/gwas/), with accession numbers GCST004750 (LUSC) and GCST004744 (LUAD).  Immunity-wide GWAS Data: Data on immune features were sourced from the GWAS catalog, covering a comprehensive dataset of 731 immune phenotypes, with accession numbers from GCST0001391 to GCST0002121.  Setting and Locations:  Data Origin: The GWAS data used in this study are derived from large-scale studies conducted in European populations.  Study Scope: While based on European data, the findings have broader implications for understanding the role of immune cells in lung cancer globally.  Relevant Dates:  Data Collection Period: The original GWAS data were collected over various periods, as detailed in the respective studies (e.g., McKay et al. 2017 for lung cancer GWAS).  Recruitment, Exposure, Follow-up:  Recruitment and Exposure: As this study is a secondary analysis of existing GWAS data, details of recruitment and exposure pertain to the original GWAS studies.  Follow-up and Data Collection: Follow-up and data collection periods are as per the original GWAS datasets, with the focus of this MR study being on the analysis of pre-existing data. |
|  | b) | Participants: Give the eligibility criteria, and the sources and methods of selection of participants. Report the sample size, and whether any power or sample size calculations were carried out prior to the main analysis |  | Eligibility Criteria and Participant Selection:  Criteria: The study utilized pre-existing GWAS datasets, hence the eligibility criteria and participant selection were as per the original GWAS studies. These studies typically include criteria based on age, health status, and specific genetic markers.  Sample Size:  GWAS Data for Lung Cancer: The GWAS data for lung cancer included 129,809 European individuals (18,699 cases and 111,110 controls) with approximately 156,688,129 variants analyzed.  Immunity-wide GWAS Data: The immune feature GWAS involved a cohort of 3,757 European individuals, with no overlapping cohorts. |
|  | c) | Describe measurement, quality control and selection of genetic variants |  | Measurement of Genetic Variants:  Data Source: The measurement of genetic variants was conducted as part of the GWAS datasets. Specific methods of genetic measurement, including SNP genotyping platforms, are detailed in the original GWAS publications.  Quality Control:  Data Filtering: The GWAS data underwent rigorous quality control measures, including filtering for call rate, minor allele frequency, and Hardy-Weinberg equilibrium, as detailed in the original GWAS methodology.  Selection of Genetic Variants:  Criteria for Instrumental Variables: In this MR study, SNPs were selected as instrumental variables based on their association with immune cell phenotypes. The selection criteria included a P-value of less than 1×10⁻⁵ from the GWAS data related to each immune trait. SNPs exhibiting linkage disequilibrium (LD) r² values greater than 0.1 within a 500 kb range were filtered out using the PLINK tool.  Instrument Strength: The strength of the selected SNPs as instrumental variables was assessed by calculating the F-statistic for each immune trait, ensuring they are strong and valid instruments for the MR analysis. |
|  | d) | For each exposure, outcome, and other relevant variables, describe methods of assessment and diagnostic criteria for diseases |  | Exposure Assessment:  Immune Cell Phenotypes: The exposure in this study consists of 731 immune cell phenotypes. These were assessed using statistical data from the GWAS catalog, with accession numbers ranging from GCST0001391 to GCST0002121. The immune phenotypes encompassed various cell types and markers, categorized into absolute cell counts, median fluorescence intensities, morphological parameters, and relative cell counts.  Outcome Assessment:  Lung Cancer Subtypes (LUSC and LUAD): The outcomes of interest are lung squamous cell carcinoma (LUSC) and lung adenocarcinoma (LUAD). These were identified using GWAS summary data for lung cancer, sourced from the GWAS Catalog with accession numbers GCST004750 (LUSC) and GCST004744 (LUAD). The diagnostic criteria for these cancer subtypes are based on established clinical and histological parameters.  Other Relevant Variables:  Confounding Factors: Potential confounders, such as smoking, rheumatoid arthritis, body mass index, and genetic predispositions, were assessed using the Phenoscanner V2 website. This assessment helped ensure that the selected SNPs for MR analysis were independent of these established risk factors. |
|  | e) | Provide details of ethics committee approval and participant informed consent, if relevant |  | Ethics Committee Approval:  Approval Details: This study is based on the analysis of pre-existing GWAS datasets. The original studies from which these data were derived obtained ethics committee approval. Details of these approvals can be found in the original GWAS publications.  Participant Informed Consent:  Consent Process: The participants in the original GWAS studies provided informed consent for their genetic data to be used in research. The process and documentation of informed consent are described in the methodology section of the original GWAS studies. |
| 5 | **Assumptions** | Explicitly state the three core IV assumptions for the main analysis (relevance, independence and exclusion restriction) as well assumptions for any additional or sensitivity analysis |  | Core IV Assumptions for Main Analysis:  Relevance: Genetic variants (IVs) are strongly associated with immune cell phenotypes.  Independence: IVs are independent of confounders affecting both exposure and outcome.  Exclusion Restriction: IVs influence the outcome only through the exposure.  Assumptions for Additional or Sensitivity Analysis:  Weighted Median Method: Assumes that at least 50% of the information comes from valid instrumental variables (IVs).  MR-Egger: Assumes balanced pleiotropic effects.  MR-PRESSO: Used for detecting and correcting outliers in instrumental variable analysis.  F-Statistics: Employed to calculate statistical power, ensuring the strength and validity of the selected genetic variants as instrumental variables.  Q-Statistic: Used to detect heterogeneity among the instrumental variables.  False Discovery Rate (FDR): Applied in multiple testing to correct for the probability of false positive findings.. |
| 6 | **Statistical methods: main analysis** | Describe statistical methods and statistics used |  |  |
|  | a) | Describe how quantitative variables were handled in the analyses (i.e., scale, units, model) |  | Handling of Quantitative Variables:  Scale and Units: The quantitative variables, primarily immune cell phenotypes, were analyzed using their respective scales and units as reported in the GWAS datasets.  Statistical Model: The study employed the inverse variance weighted (IVW) method as the primary model for Mendelian Randomization analysis. This method combines the Wald estimates for each SNP to estimate the overall effect of the exposure on the outcome.  Data Transformation: No data transformation was performed for the analysis. |
|  | b) | Describe how genetic variants were handled in the analyses and, if applicable, how their weights were selected |  | Handling of Genetic Variants:  Variant Selection: In this study, SNPs were selected as instrumental variables based on a P-value of less than 1×10⁻⁵ from the GWAS data related to each immune trait. SNPs exhibiting linkage disequilibrium (LD) r² values greater than 0.1 within a 500 kb range were filtered out using the PLINK tool.  Weight Selection: For the inverse variance weighted (IVW) method, the weights of each SNP were determined by the precision of their estimates, typically using the inverse of the variance of the Wald ratio for each SNP. |
|  | c) | Describe the MR estimator (e.g. two-stage least squares, Wald ratio) and related statistics. Detail the included covariates and, in case of two-sample MR, whether the same covariate set was used for adjustment in the two samples |  | MR Estimator and Statistics:  MR Estimator: The primary MR estimator used in this study was the inverse variance weighted (IVW) method. This approach combines the Wald ratios of individual SNPs to estimate the overall causal effect.  Related Statistics: Additional statistical methods for sensitivity analysis, such as MR-Egger regression and the weighted median estimator, were likely employed to assess the robustness of the results and to test for potential pleiotropic effects.  Covariates:  No adjustment was made for covariates |
|  | d) | Explain how missing data were addressed |  | Addressing Missing Data:  The study did not specifically mention the handling of missing data. It can be assumed that the analysis was based on complete cases |
|  | e) | If applicable, indicate how multiple testing was addressed |  | Addressing Multiple Testing:  The study applied False Discovery Rate (FDR) correction to address the issue of multiple testing, particularly relevant when analyzing a large number of genetic variants. This method adjusts the p-values to reduce the risk of type I errors (false positives) in the context of multiple comparisons. |
| 7 | **Assessment of assumptions** | Describe any methods or prior knowledge used to assess the assumptions or justify their validity |  | The study employed F-statistics and MR-Egger intercept to assess the validity of the core assumptions of Mendelian Randomization. |
| 8 | **Sensitivity analyses and additional analyses** | Describe any sensitivity analyses or additional analyses performed (e.g. comparison of effect estimates from different approaches, independent replication, bias analytic techniques, validation of instruments, simulations) |  | Initially, the Q-test method was utilized to evaluate potential heterogeneity among individual IVs, and p-value less than 0.05 from the Cochran Q test is considered indicative of heterogeneity in the results. Subsequently, the MR‐Egger intercept test was applied to estimate horizontal pleiotropy, guaranteeing that genetic variation has an independent relationship with both the exposure and outcome. We used MR-PRESSO to re-examine the presence of heterogeneous SNPs. Additionally, we conducted a leave-one-out (LOO) analysis, assessing whether the results were significantly influenced by individual SNPs by sequentially dropping each SNP and then performing MR analysis.  Replication and Meta-Analysis: The study involved independent replication using the Transdisciplinary Research Into Cancer of the Lung (TRICL) data and a meta-analysis to validate the findings and enhance their robustness. |
| 9 | **Software and pre-registration** |  |  |  |
|  | a) | Name statistical software and package(s), including version and settings used |  | All statistical analyses were performed using R version 4.3.0. Specifically, for MR analysis, we employed the MendelianRandomization package0.9.0, TwoSampleMR0.5.7, MRPRESSO package 1.0. |
|  | b) | State whether the study protocol and details were pre-registered (as well as when and where) |  | The study did not involve pre-registration of the study protocol. |
|  | **RESULTS** |  |  |  |
| 10 | **Descriptive data** |  |  |  |
|  | a) | Report the numbers of individuals at each stage of included studies and reasons for exclusion. Consider use of a flow diagram |  | The methodology section of this paper provides the number of the sample population, but information about the population is not provided in the results section. Information on the excluded population and reasons for exclusion are not provided, nor is a flow diagram used. |
|  | b) | Report summary statistics for phenotypic exposure(s), outcome(s), and other relevant variables (e.g. means, SDs, proportions) |  | The example text provides relevant information in Supplementary Tables S1-S2, which present information about the study subjects. |
|  | c) | If the data sources include meta-analyses of previous studies, provide the assessments of heterogeneity across these studies |  | Assessments of Heterogeneity:  Meta-Analysis Inclusion: The study included meta-analyses for both lung squamous cell carcinoma (LUSC) and lung adenocarcinoma (LUAD) to validate the causal relationships with specific immune phenotypes.  Heterogeneity Assessments: Heterogeneity across the studies included in the meta-analyses was assessed. The results indicated no significant heterogeneity among the studies, suggesting consistency in the findings across different datasets.As shown in Supplementary Tables S8 |
|  | d) | For two-sample MR:  i.  Provide justification of the similarity of the genetic variant-exposure associations between the exposure and outcome samples  ii.  Provide information on the number of individuals who overlap between the exposure and outcome studies |  | No report |
| 11 | **Main results** |  |  |  |
|  | a) | Report the associations between genetic variant and exposure, and between genetic variant and outcome, preferably on an interpretable scale |  | No report |
|  | b) | Report MR estimates of the relationship between exposure and outcome, and the measures of uncertainty from the MR analysis, on an interpretable scale, such as odds ratio or relative risk per SD difference |  | Lung Adenocarcinoma (LUAD): The study did not find statistically significant differences between immune cell phenotypes and LUAD.  Lung Squamous Cell Carcinoma (LUSC): Our LUSC study revealed associations with risk: CD28 on resting CD4 regulatory T cells (OR = 1.11, 95% CI = 1.06–1.16, p = 1.70E-05, PFDR = 1.2E-02) and CD45RA+ CD28- CD8+ T cell %T cell (OR = 1.00, 95% CI = 1.00–1.00, p = 2.60E-04, PFDR =3.10E-02). Additionally, we identified an immunophenotype, CCR2 on monocytes (OR = 0.93, 95% CI: 0.90–0.97, p = 8.10E-5, FDR = 1.6E-02), exhibiting protective effects against LUSC susceptibility. |
|  | c) | If relevant, consider translating estimates of relative risk into absolute risk for a meaningful time period |  | No report |
|  | d) | Consider plots to visualize results (e.g. forest plot, scatterplot of associations between genetic variants and outcome versus between genetic variants and exposure) |  | The study includes various plots to visualize the results of the MR analysis. These may include forest plots for displaying MR estimates for each immune phenotype associated with lung cancer subtypes (LUSC and LUAD), and scatterplots to illustrate the associations between genetic variants and lung cancer subtypes versus the associations between genetic variants and immune cell phenotypes. |
| 12 | **Assessment of assumptions** |  |  |  |
|  | a) | Report the assessment of the validity of the assumptions |  | The example text reports the results of the assessment of the validity of relevant assumptions in multiple places throughout the main text and the appendices, such as in Supplementary Tables 3-4, S6-7 [15]. It reports the statistical power of the instrumental variables in each association, represented by F-statistic values. The Q-statistic is used to detect heterogeneity in the statistical model, assessing its stability. |
|  | b) | Report any additional statistics (e.g., assessments of heterogeneity across genetic variants, such as *I^2^*, Q statistic or E-value) |  | The study includes assessments of heterogeneity across the genetic variants used as instrumental variables, utilizing the Q statistic (Q test).,such as in Supplementary Tables 3-4, S6-7. |
| 13 | **Sensitivity analyses and additional analyses** |  |  |  |
|  | a) | Report any sensitivity analyses to assess the robustness of the main results to violations of the assumptions |  | Subsequent analysis involved a further evaluation of these seven immune cell traits. We excluded four immune cells - SSC-A on lymphocyte, HLA DR on CD33- HLA, CD20 on IgD- CD24- B cell, and HLA DR on Dendritic Cell - based on not meeting the criteria of Q-test method p<0.05, or MR-Egger intercept test p<0.05, or MR-PRESSO method p<0.05(Supplementary Table S5).  The refined analysis focused on three types of immune cells, comprising two from the Treg panel and one from the cDC panel. Our LUSC study revealed associations with risk: CD28 on resting CD4 regulatory T cells (OR = 1.11, 95% CI = 1.06–1.16, p = 1.70E-05, PFDR = 1.2E-02) and CD45RA+ CD28- CD8+ T cell %T cell (OR = 1.00, 95% CI = 1.00–1.00, p = 2.60E-04, PFDR =3.10E-02). Additionally, we identified an immunophenotype, CCR2 on monocytes (OR = 0.93, 95% CI: 0.90–0.97, p = 8.10E-5, FDR = 1.6E-02), exhibiting protective effects against LUSC susceptibility.  In summury, The IVW estimates for the three selected immune cells were significant, maintained significance after FDR correction, and were consistent in direction and magnitude (Fig.3). The Cochran Q Test (p> 0.05) and MR-Egger intercept test (p> 0.05) indicated no heterogeneity or pleiotropy. Similarly, MR-PRESSO results, after outlier removal, did not suggest the presence of heterogeneous SNPs（Table 1）. The LOO analysis further supported the reliability of our MR estimation, as shown in Supplementary Figure S1. These findings led us to consider these three immune cells as prime candidates for further analysis (Supplementary Fig. S2). |
|  | b) | Report results from other sensitivity analyses or additional analyses |  | No report |
|  | c) | Report any assessment of direction of causal relationship (e.g., bidirectional MR) |  | No report |
|  | d) | When relevant, report and compare with estimates from non-MR analyses |  | No report |
|  | e) | Consider additional plots to visualize results (e.g., leave-one-out analyses) |  | The study includes leave-one-out analysis plots, as shown in the Supplementary Fig. S2. within the article |
|  | **DISCUSSION** |  |  |  |
| 14 | **Key results** | Summarize key results with reference to study objectives |  | Our study suggests that CD28 on resting CD4 regulatory T cells and CD45RA+ CD28- CD8+ T cell %T cell increases the risk of LUSC, while a phenotype of CCR2 on monocytes is associated with a reduced risk of LUSC. However, we did not find any immune cells with a causal relationship in LUAD. Our conclusions were further reinforced by replication and meta-analysis, and subsequently, our estimates remained significant after excluding SNPs associated with confounding factors. |
| 15 | **Limitations** | Discuss limitations of the study, taking into account the validity of the IV assumptions, other sources of potential bias, and imprecision. Discuss both direction and magnitude of any potential bias and any efforts to address them |  | Nevertheless, the study has some limitations. First, the analysis is based on publicly available GWAS data, which only represents European populations. Therefore, caution should be taken when extrapolating the results of this study to other ethnic groups. Second, as mentioned above, our observations on CCR2+ monocytes differ from current literature viewpoints. Such discrepancies may arise due to variations in sample sources, experimental conditions, or statistical methods. Biological complexity in dynamic systems of the tumor microenvironment may also be a relevant factor. Thus, investigating the precise role of circulating CCR2+ monocytes in LUSC risk becomes necessary, considering the current controversy. Finally, our study primarily examines specific immune cell traits, but we cannot disregard the impacts of other cell types or factors that may impact the risk of LUSC. For instance, certain tumor-infiltrating myeloid cell subsets, m6A regulation, genomic alterations, and specific somatic mutations can play pivotal roles in regulating the interactions between various immune cells and influence tumor development . Therefore, while our research lays the groundwork, more comprehensive understandings of these factors and their interactions are required. |
| 16 | **Interpretation** |  |  |  |
|  | a) | Meaning: Give a cautious overall interpretation of results in the context of their limitations and in comparison with other studies |  | The example text dedicates the largest section to discussing this item, providing a reasonable interpretation of the MR results by comparing them with multiple published studies. |
|  | b) | Mechanism: Discuss underlying biological mechanisms that could drive a potential causal relationship between the investigated exposure and the outcome, and whether the gene-environment equivalence assumption is reasonable. Use causal language carefully, clarifying that IV estimates may provide causal effects only under certain assumptions |  | The article has explored the possible biological mechanisms. |
|  | c) | Clinical relevance: Discuss whether the results have clinical or public policy relevance, and to what extent they inform effect sizes of possible interventions |  | In summary, we performed a two-sample Mendelian randomization study to investigate the causal connections between different immune phenotypes and LUSC or LUAD. Our analysis demonstrated that the causal relationship is more pronounced in LUSC and three immune cell types correlated with LUSC susceptibility, while the association with LUAD is statistically insignificant. These findings enhance our comprehension of the complex interplay between the immune system and lung cancers. This study yields new perspectives into managing cancer risk, potentially providing more precise therapeutic alternatives for individuals with lung cancer, and furnishing valuable information for future scientific research. |
| 17 | **Generalizability** | Discuss the generalizability of the study results (a) to other populations, (b) across other exposure periods/timings, and (c) across other levels of exposure |  | The text reports in the discussion that "Furthermore, while this study's limitation to a European ancestry population may reduce potential biases from population stratification, it limits the generalizability of the MR results to other populations." |
|  | **OTHER INFORMATION** |  |  |  |
| 18 | **Funding** | Describe sources of funding and the role of funders in the present study and, if applicable, sources of funding for the databases and original study or studies on which the present study is based |  | This study was made possible through the generous support from the Natural Science Foundation of China (grant no. 82060078) and the Guangxi Key Research and Development Program (Guike AB23026006). |
| 19 | **Data and data sharing** | Provide the data used to perform all analyses or report where and how the data can be accessed, and reference these sources in the article. Provide the statistical code needed to reproduce the results in the article, or report whether the code is publicly accessible and if so, where |  | All data used in this study are publicly available. No human subject approvals were necessary to conduct these analyses. All the data can be found from the GWAS directory (https://gwas.mrcieu.ac). The serial numbers of the immune cells are from GCST0001391 to GCST0002121, respectively. For the LUSC and LUAD data used for the primary analysis, the serial number is GCST004750 and GCST004744, and the LUSC and LUAD serial numbers used for the replication and meta-analysis are ieu-a-989 and ieu-a-984 |
| 20 | **Conflicts of Interest** | All authors should declare all potential conflicts of interest |  | The authors declare that they have no competing interests. |

This checklist is copyrighted by the Equator Network under the Creative Commons Attribution 3.0 Unported (CC BY 3.0) license.

1. Skrivankova VW, Richmond RC, Woolf BAR, Yarmolinsky J, Davies NM, Swanson SA, et al. Strengthening the Reporting of Observational Studies in Epidemiology using Mendelian Randomization (STROBE-MR) Statement. JAMA. 2021;under review.

2. Skrivankova VW, Richmond RC, Woolf BAR, Davies NM, Swanson SA, VanderWeele TJ, et al. Strengthening the Reporting of Observational Studies in Epidemiology using Mendelian Randomisation (STROBE-MR): Explanation and Elaboration. BMJ. 2021;375:n2233.
