# Supplementary material for: Associations between immune cell phenotypes and lung cancer subtypes: insights from mendelian randomization analysis
Source: BMC Pulm Med. 2024 May 16;24:242. doi: 10.1186/s12890-024-03059-w (PMC11100125; doi:10.1186/s12890-024-03059-w)
Supplement: Supplementary file 6 — Supplementary Material 6 [file 12890_2024_3059_MOESM6_ESM.docx]

**Treg panel:** Tregs were identified on the basis of high expression of CD25 and low expression of CD127 surface antigens (CD25hiCD127lo) and further subdivided into activated (CD25+++CD45RA−), resting (CD25++CD45RA+) and secreting (CD25++CD45RA−).

**T lymphocytes:** T lymphocytes were split into six subsets based on the expression of the CD4 and CD8 markers: CD4−CD8− (DN), CD4−CD8dim (CD8dim), CD4−CD8bright (CD8br), CD4+CD8br (DP), CD4+CD8dim, CD4+CD8− (CD4+). The HLA DR positivity of CD4+ and CD8br T cells and NK cells was considered as an activation marker.

**Monocyte panel:** Monocytes were identified on the basis of morphological parameters and HLA DR positivity. Monocytes were then subdivided into classical (CD14+CD16−), non-classical (CD14−CD16+) and intermediate (CD14+CD16+).

**Maturation stages of T-cell panel**：The maturation status of CD4+, CD8br and CD4−CD8− T lymphocytes was assessed on the basis of the expression of CD45RA and CCR7. Naive (CD45RA+CCR7+), central memory (CCR7+CD45RA−), effector memory (CD45RA−CCR7−) and terminally differentiated (CCR7−CD45RA+) maturation stages were identified.

**DC panel:** DCs were identified on the basis of their positivity for HLA DR and their negativity for the lineage cocktail (Lin) targeting CD3, CD14, CD16, CD19, CD20 and CD56 markers. The DCs were subdivided into myeloid (CD11c+) and plasmacytoid (CD123+) cells. Their maturation and activation status were ascertained by the adhesion molecule CD62L and the co-stimulatory molecules CD80 and CD86. In addition, monocytes were morphologically ascertained and analyzed for HLA DR, CD62L and CD11c expression.

**B-cell panel:** CD24 versus CD38 classification identified transitional (CD24+CD38hi), memory (CD24+CD38−/dim) and naive mature (CD24−CD38−/dim) subsets. CD27 versus IgD classification discriminated switched memory (CD27+IgD−), unswitched memory (CD27+IgD+), naive (CD27−IgD+) and CD27−IgD− B cells. IgD versus CD38 classification, also known as Bm1–Bm5 classification, distinguished six B cell subsets: Bm1 (IgD+CD38−) mainly virgin naive cells; Bm2 (IgD+CD38dim) activated naive cells; Bm2′ (IgD+CD38br) pre-germinal center cells; Bm3–Bm4 (IgD−CD38br) centroblasts and centrocytes present in germinal center cells but very low/absent in blood; early Bm5 (IgD−CD38dim); and late Bm5 (IgD−CD38−) memory cells. CD24 versus CD27 classification identified CD24+CD27+ memory cells. IgD versus CD24 classification subdivided B cells into four subsets: IgD+CD24+; IgD−CD24+; IgD−CD24; and IgD+CD24−. CD20 versus CD38 discriminated plasma blasts/plasma cells (as CD20−CD38hi) and CD20−CD38− cells.

**Myeloid cell panel:** The fluorescent intercalator 7-aminoactinomycin D was used to recognize and exclude dead cells. In parallel, a cocktail including CD19, CD20 and CD3 antibodies was used to remove lymphoid cells. The resulting myeloid-enriched cells were subdivided on the basis of CD14 high positivity (corresponding to classical monocytes) and into five subsets based on CD33 and HLA DR expression. CD11b and CD66b antibodies were used for additional sub-characterization. The CD33dim HLA DR− were subdivided into: granulocytic myeloid-derived suppressor cells (MDSCs), based on the high positivity for CD66b cells; immature MDSCs, which are negative for CD11b; and basophils, which are positive for CD11b. Monocytic MDSCs were identified on the basis of their high positivity for CD14 and CD33 and weak positivity for HLA DR. Finally, hematopoietic stem cells were identified as CD34+CD45dim.
